# *PARK2* Mutation Causes Metabolic Disturbances and Impaired Survival of Human iPSC-Derived Neurons

**DOI:** 10.3389/fncel.2019.00297

**Published:** 2019-07-05

**Authors:** Helle Bogetofte, Pia Jensen, Matias Ryding, Sissel I. Schmidt, Justyna Okarmus, Louise Ritter, Christina S. Worm, Michaela C. Hohnholt, Carla Azevedo, Laurent Roybon, Lasse K. Bak, Helle Waagepetersen, Brent J. Ryan, Richard Wade-Martins, Martin R. Larsen, Morten Meyer

**Affiliations:** ^1^Department of Neurobiology Research, Institute of Molecular Medicine, University of Southern Denmark, Odense, Denmark; ^2^Oxford Parkinson’s Disease Centre, Medical Sciences Division, Department of Physiology, Anatomy and Genetics, University of Oxford, Oxford, United Kingdom; ^3^Department of Biochemistry and Molecular Biology, University of Southern Denmark, Odense, Denmark; ^4^Department of Drug Design and Pharmacology, Faculty of Health and Medical Sciences, University of Copenhagen, Copenhagen, Denmark; ^5^Department of Experimental Medical Science, Wallenberg Neuroscience Center, Lund University, Lund, Sweden; ^6^Brain Research – Inter-Disciplinary Guided Excellence, University of Southern Denmark, Odense, Denmark

**Keywords:** Parkinson’s, proteomics, oxidative stress, metabolism, survival, mitochondria

## Abstract

The protein parkin, encoded by the *PARK2* gene, is vital for mitochondrial homeostasis, and although it has been implicated in Parkinson’s disease (PD), the disease mechanisms remain unclear. We have applied mass spectrometry-based proteomics to investigate the effects of parkin dysfunction on the mitochondrial proteome in human isogenic induced pluripotent stem cell-derived neurons with and without *PARK2* knockout (KO). The proteomic analysis quantified nearly 60% of all mitochondrial proteins, 119 of which were dysregulated in neurons with *PARK2* KO. The protein changes indicated disturbances in oxidative stress defense, mitochondrial respiration and morphology, cell cycle control, and cell viability. Structural and functional analyses revealed an increase in mitochondrial area and the presence of elongated mitochondria as well as impaired glycolysis and lactate-supported respiration, leading to an impaired cell survival in PARK2 KO neurons. This adds valuable insight into the effect of parkin dysfunction in human neurons and provides knowledge of disease-related pathways that can potentially be targeted for therapeutic intervention.

## Introduction

Parkinson’s disease (PD) is an incurable neurodegenerative disorder characterized by the progressive loss of midbrain dopaminergic neurons and subsequent striatal dopamine depletion. Key contributions to the pathogenesis of both sporadic and familial PD come from mitochondrial dysfunction and oxidative stress (Abou-Sleiman et al., 2006; Ryan et al., 2015). This is supported by studies demonstrating that toxins inhibiting the mitochondrial respiratory chain complex I produce PD pathogenesis in both humans and animal models by elevating the production of reactive oxygen species (ROS) and causing dopaminergic cell death (Langston et al., 1983; Langston, 1996). Importantly, decreased activity of complex I and increased oxidative damage have been detected in substantia nigra of sporadic PD patients (Dexter et al., 1989; Parker et al., 1989; Schapira et al., 1990; Alam et al., 1997).

Mutations in the genes *PARK2* (parkin), *PARK6* (PINK1), and *PARK7* (DJ-1), which are all important for mitochondrial function, cause autosomal recessive familial PD, with *PARK2* mutations being the most common (Abou-Sleiman et al., 2006). *PARK2* encodes the ubiquitin E3 ligase, parkin, which is recruited to damage mitochondria by PINK1. Phosphorylation of ubiquitin and parkin by PINK1 is required for parkin recruitment and activity (Kazlauskaite et al., 2014). When activated, parkin ubiquitinates target mitochondrial proteins, marking them and the mitochondria for degradation through the autophagy–lysosome pathway (ALP), a process known as mitophagy (Clark et al., 2006; Narendra et al., 2008). Besides its role in mitochondrial quality control, parkin appears to be involved in numerous other functions, including cytoskeletal stability, cell mitosis, and cell survival, where its role is less well described (Moore, 2006). The study of parkin dysfunction in PD pathogenesis is, however, impeded by the fact that genetic parkin knockout (KO) rodents show only minor disease phenotypes and limited pathology (Kitada et al., 2009; Oliveras-Salvá et al., 2011).

Instead induced pluripotent stem cells (iPSCs) derived from patients with familial PD or cells with *PARK2* mutations introduced by genome editing have enabled investigations of parkin dysfunction in human dopaminergic neurons *in vitro*. PD patient iPSC-derived neurons with *PARK2* mutations show increased oxidative stress, α-synuclein accumulation, and disturbances in mitochondrial morphology and function (Imaizumi et al., 2012; Jiang et al., 2012; Chung et al., 2016). However, the cellular changes and sequence of events leading from disruption of parkin function to disease development remain largely unknown.

Mass spectrometry-based proteomics enable the unbiased characterization of changes in large numbers of proteins simultaneously. Such methods have been applied to cell and animal models of parkin and PINK1 dysfunction revealing changes in proteins involved in oxidative stress, synaptic plasticity, protein folding, and energy metabolism (Ozgul et al., 2015; Triplett et al., 2015). In addition, several proteomics studies have specifically aimed at identifying and quantifying changes in mitochondrial proteins in various PD models, however, with limited commonality in their findings, perhaps due to variability between the different models (Aroso et al., 2016).

We report for the first time the investigation of mitochondria-specific proteomic and phospho-proteomic changes caused by parkin dysfunction in human iPSC-derived neurons. Our analysis identified and quantified more than half of all reported mitochondrial proteins and determined large numbers of dysregulated proteins in *PARK2* KO neurons compared to healthy isogenic controls. Among the affected mitochondrial proteins were key components in the cellular defense against oxidative stress as well as in glycolysis and lactate–pyruvate metabolism, which was confirmed by a functional impairment of glycolysis and lactate-supported respiration. Such changes may lead to mitochondrial energy deficits, increased susceptibility to oxidative stress, and cell death, which was seen for neurons with PARK2 KO in the present study.

## Materials and Methods

### Ethics Statement

All use of human stem cells was performed in accordance with the Danish national regulations, the ethical guidelines issued by the Network of European CNS Transplantation and Restoration (NECTAR) and the International Society for Stem Cell Research (ISSCR).

### NSCs Culture and Differentiation

The control and *PARK2* KO iPSC lines and corresponding NSC derivatives were obtained from XCell Science Inc. (Novato, CA, United States) (Shaltouki et al., 2015). iPSCs and NSCs were characterized and propagated according to standard protocols. iPSC differentiation to NSCs was performed by XCell Science Inc. using a 14-day protocol (Swistowski et al., 2009). Dopaminergic differentiation was achieved by culturing NSCs in DOPA Induction and Maturation Medium (XCell Science Inc., Novato, CA, United States) according to manufacturer instructions for at least 25 days from NSC stage (39 days from iPSC stage). See the Supplementary Information for detailed procedures.

### Mitochondrial Enrichment

Mitochondria were purified from day 25 *PARK2* KO and control neuronal cultures from three independent differentiations (39 days differentiation from iPSC stage). Briefly, the cells were collected on ice in phosphate-buffered saline (PBS, ThermoFisher) with protease (Complete Tablets, Roche) and phosphatase inhibitors (PhosSTOP Tablets, Roche) and stored at -20°C. The QProteome Mitochondria Isolation Kit (Qiagen #37612) was used according to the manufacturer’s instruction for standard preparation.

### Lysis, Reduction, and Enzymatic Digestion

Samples were incubated for 2 h in 10 μl lysis buffer consisting of 6 M urea, 2 M thiourea, and 10 mM DTT (all Sigma). Thereafter, samples were dilutes 10 times in 20 mM triethylammonium bicarbonate buffer (TEAB, Sigma), pH 7.5, and sonicated two times 10 s on ice followed by alkylation with 20 mM Iodacetamide (Sigma) for 30 min in the dark. Protein concentration was measured by Qubit^®^ according to the manufacturer’s instruction. Samples were digested with 1 μg trypsin (Sigma) per 50 μg protein at room temperature (RT).

### Tandem Mass Tag Labeling and Enrichment of Phosphorylated Peptides

Forty-five micrograms of each sample were labeled with tandem mass tag (TMT) Sixplex Isobaric label Reagents (ThermoFisher) according to the manufacturer’s instructions. Efficient labeling was confirmed by MALDI, the ratios adjusted, and the labeled peptides mixed 1:1:1:1:1:1 and dried by vacuum centrifugation.

Phospho-peptide enrichment was essentially performed as earlier described (Engholm-Keller et al., 2012). See the Supplementary Information for detailed procedure.

### Hydrophobic Interaction Liquid Chromatography (HILIC) and High pH Fractionation

Mono-phosphorylated and non-modified peptides were fractionated to reduce sample complexity by HILIC as described previously (Engholm-Keller et al., 2012). Detailed description can be found in the Supplementary Information. To increase the coverage, high pH fractionation was also performed using approximately 50 μg peptide of the non-modified peptide sample. Briefly, the sample was dissolved in 1% ammonium (NH_3_, Sigma), pH 11, and loaded on a R2/R3 column equilibrated with 0.1% NH_3_. The peptides were eluted in a stepwise fashion using a gradient of 5–60% ACN/0.1% NH_3_. All fractions were dried by vacuum centrifugation.

### Reversed-Phase NanoLC–ESI–MS/MS

The samples were resuspended in 0.1% formic acid (FA) and loaded onto a two-column EASY-nLC System (Thermo Scientific). The pre-column was a 3 cm long fused silica capillary (100 μM inner diameter) with a fritted end and in-house packed with ReproSil-Pur C18 AQ 5 μm (Dr. Maisch GmbH) whereas the analytical column was a 17 cm long fused silica capillary (75 μm inner diameter) and packed with ReproSil-Pur C18 AQ 3 μm reversed-phase material (Dr. Maisch GmbH).

The peptides were eluted with an organic solvent gradient from 100% phase A (0.1% FA) to 34% phase B (95% ACN, 0.1% FA) at a constant flow-rate of 250 nl/min. Depending on the samples based on the HILIC, the gradient was from 1 to 30% solvent B in 60 or 90 min, 30–50% solvent B in 10 min, 50–100% solvent B in 5 min and 8 min at 100% solvent B.

The nLC was online connected to a QExactive HF Mass Spectrometer (Thermo Scientific) operated at positive ion mode with data-dependent acquisition. The Orbitrap acquired the full MS scan with an automatic gain control (AGC) target value of 3 × 10^6^ ions and a maximum fill time of 100 ms. Each MS scan was acquired at high-resolution [120,000 full width half maximum (FWHM)] at *m*/*z* 200 in the Orbitrap with a mass range of 400–1400 Da. The 12 most abundant peptide ions were selected from the MS for higher energy collision-induced dissociation (HCD) fragmentation (collision energy: 34 V). Fragmentation was performed at high resolution (60,000 FWHM) for a target of 1 × 10^5^ and a maximum injection time of 60 ms using an isolation window of 1.2 *m*/*z* and a dynamic exclusion. All raw data were viewed in Thermo Xcalibur v3.0.

### Mass Spectrometry Data Analysis

The raw data were processed using Proteome Discoverer (v2.1, ThermoFisher) and searched against the Swissprot human database using an in-house Mascot server (v2.3, Matrix Science Ltd.) and the Sequest HT search engine.

Database searches were performed with the following parameters: precursor mass tolerance of 10 ppm, fragment mass tolerance of 0.02 Da (HCD fragmentation), TMT 6-plex (Lys and N-terminal) as fixed modifications, and a maximum of two missed cleavages for trypsin. Variable modifications were carbamidomethylation of alkylated Cys and N-terminal acetylation along with phosphorylation of Ser/Thr/Tyr for the phosphorylated group. Only peptides with up to a *q*-value of 0.01 (Percolator), Mascot rank 1, and cut-off value of Mascot score >15 were considered for further analysis. Only proteins with more than one unique peptide were considered for further analysis in the non-modified group.

The mass spectrometry proteomics data have been deposited to the ProteomeXchange Consortium via the PRIDE partner repository with the dataset identifier PXD008894 (Vizcaíno et al., 2016).

Pathway analysis on proteomics data was performed using String 10.0, ProteinCenter (ThermoFisher) and the ingenuity pathway analysis (IPA) software (QIAGEN).

### Immunofluorescence and Western Blotting

Immunofluorescence and Western blotting were performed using standard methods. Detailed descriptions can be found in the Supplementary Information. Primary antibodies were used in the following concentrations:

For immunofluorescence: rabbit anti-tyrosine hydroxylase (TH, Millipore #AB152) 1:600, mouse anti-TH (Millipore #AB5280) 1:600, mouse anti-microtubule-associated protein 2a+b (MAP2, Sigma #M1406) 1:2000, mouse anti-Ki67 (BD Pharmingen #550609) 1:500, goat anti-Annexin V (GeneCopoeia #A037) 1:200, mouse anti-synaptophysin (Sigma # S5768) 1:200, and rabbit anti-TOM20 (Santa Cruz #SC-11415) 1:1000.

For Western blotting: Mouse anti-β-actin-HRP (Abcam #49900) 1:50,000, rabbit anti-BAX (Cell Signaling #5023) 1:1000, rabbit anti-COXIV (Abcam #16056) 1:2000, rabbit anti-DJ-1 (Abcam #18257) 1:1000, mouse anti-lactate dehydrogenase (LDH) (Santa Cruz #133123) 1:100, rabbit anti-MIEF1 (Abcam #89944) 1:1000, mouse anti-Parkin (Cell Signaling #4211) 1:1000, rabbit anti-SOD1 (Abcam #16831) 1:2000, mouse anti-TH (Millipore #AB5280) 1:600, anti-TOM20 (Santa Cruz #11415) 1:1000, rabbit anti-voltage-dependent anion-selective channel 1 (VDAC1) (Abcam #154856) 1:1000, and rabbit anti-14-3-3𝜀 (Cell Signaling #9635) 1:1000.

Fluorescence images were acquired on an FV1000MPE confocal microscope (Olympus). Analysis of mitochondrial morphology and numbers was performed in ImageJ by converting TOM20 images to binary format and using the “analyse particles” function. For mitochondrial morphology the following criteria were applied after calculating the ratio of pixels^2^ to μm^2^: Large, elongated: size = 0.71–3.50 μm^2^, circularity = 0.01–0.50. Large, round: size = 0.71–3.50 μm^2^, circularity = 0.50–1.00. Small, elongated: size = 0.15–0.70 μm^2^, circularity = 0.01–0.50. Small, round: size = 0.15–0.70 μm^2^, circularity = 0.50–1.00. Supplementary Figure S4 shows an example of the analysis and the four categories of mitochondria.

Area and numbers of mitochondria were normalized to total cell numbers as quantified by CellProfiler analysis for DAPI+ nuclei (Carpenter et al., 2006; Schneider et al., 2012).

### Oxygen Consumption Rate Analysis

On differentiation day 18 (32 from iPSC stage), cells were replated onto poly-L-ornithine (Sigma) and laminin (Life Tech) coated Seahorse 96-well plates (Agilent Tech.) at a density of 200,000 cells/well. Four wells with no cells were used as background control in the OCR and extracellular acidification rate (ECAR) analysis. Oxygen consumption rate **(OCR)** and ECAR were measured on differentiation day 25 (from the NSC stage) on the Seahorse XFe 96 analyzer using the Seahorse XF Mito Stress Test Kit (Agilent Tech.) according to the manufacturer’s instructions. Briefly, the cells were washed once and incubated in XF Base Medium (Agilent Tech.) supplemented with either 2.5 mM glucose (Sigma) or 2.5 mM lactate (Sigma) 37°C for 5 min in a non-CO_2_ incubator prior to analysis. After equilibration in the XFe 96 analyzer the following measurements were acquired: three baseline readings, two following 2 μM oligomycin treatment, two following 2 μM FCCP treatment, and three following 0.5 μM rotenone/antimycin treatment. The background corrected OCR and ECAR readings were normalized to protein levels per well as measured by Pierce Bicinchoninic Acid (BCA) Protein Assay (ThermoFisher) and these values were normalized to the average basal level of the control in each independent experiment.

### Cell Viability and Death

Cell viability was measured by Trypan blue staining of cells in suspension following 5 min accutase treatment at differentiation days 0, 5, 10, and 25 from the NSC stage.

The nuclear morphology was quantified by counting the number of fragmented and pyknotic nuclei on DAPI-stained neuronal cultures as a proportion of total cells per field of view.

Lactate dehydrogenase release was quantified at differentiation day 25 as a measure of cell death applying the CytoTox 96 Non-Radioactive Cytotoxicity Assay (Promega) according to manufacture instructions with a cell density of 20,000 per well plated at day 18. Values were normalized to protein concentrations to account for differences in cell numbers per well.

### Statistical Analysis

An in house R-tool was applied to perform paired Limma test (moderated *t*-test) with correction for multiple testing to allow for the detection of significant protein and PTM level changes in the proteomic dataset (Storey, 2002; Schwämmle et al., 2013). Analyses of functional assays were performed in Graphpad Prism version 5.0 (GraphPad Software, United States) using two-tailed Student’s *T*-tests or one-way ANOVA with Tukey’s *post hoc* test where appropriate. Results are expressed as mean ± SEM, and *P*-values < 0.05 were considered statistically significant.

## Results

### Mitochondrial-Enriched Proteomics Quantify More Than Half of Known Mitochondrial Proteins in Control and *PARK2* KO Neurons

**FIGURE 1:**
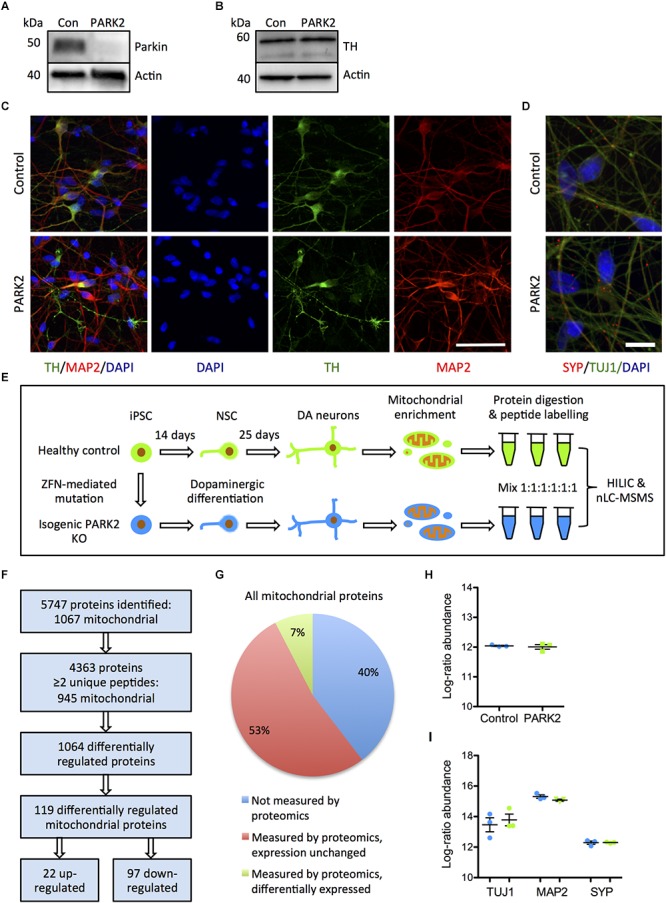
Proteomic analysis of mitochondrial changes in PARK2 knockout (KO)-induced pluripotent stem cell (iPSC)-derived neurons. **(A,B)** Western blotting for **(A)** parkin protein verifying the *PARK2* KO and **(B)** tyrosine hydroxylase (TH) indicating comparable dopaminergic (DA) differentiation. **(C)** Immunofluorescence staining of control and *PARK2* KO neurons on differentiation day 25 showed no differences in levels of TH (green) and microtubule-associated protein 2 (MAP2, red) positive neurons. Scale bar = 50 μm. **(D)** Immunofluorescence staining for synaptophysin (SYP) and β-III-tubulin (TUJ1) confirmed the presence of mature *PARK2* KO and control neurons. Scale bar = 25 μm. **(E)** Schematic illustration of the experimental set-up: *PARK2* KO iPSCs created by zinc finger nucleases (ZFNs) and the isogenic healthy control iPSC line were differentiated to neural stem cells (NSCs) and onward to DA neurons. Mitochondrial enrichment was performed on cell lysates from three independent differentiations. Proteins were digested to peptides, which were labeled and mixed in 1:1:1:1:1:1 ratio prior to hydrophilic interaction liquid chromatography (HILIC) and nano-liquid chromatography tandem mass spectrometry analysis (nLC-MSMS). **(F)** Overview of the identified proteins: 945 mitochondrial proteins were identified by two or more peptides and levels of 119 of these were significantly altered in *PARK2* KO neurons. **(G)** The analysis identified 60% of all mitochondrial proteins listed in the Gene Ontology (GO) Database (GO term: mitochondrion, organism: homo sapiens, type: protein), 7% of these were differentially expressed in *PARK2* KO neurons. **(H,I)** The average proteomics-identified abundances (log ratio) of **(H)** all mitochondrial proteins as well as **(I)** the neuronal markers TUJ1, MAP2, and SYP were similar between control and *PARK2* KO neurons. Mean ± SEM, *n* = 3 independent differentiations.

To explore the effects of parkin dysfunction, we utilized two isogenic iPSC lines created from a healthy control iPSC line, where deletions have been introduced in exon 2 on both alleles of the *PARK2* gene by genome editing (Shaltouki et al., 2015). The *PARK2* KO was confirmed by Western blotting for parkin protein (Figure 1A). We have previously demonstrated that the *PARK2* KO and isogenic control NSCs equally can be differentiated into neuronal cultures with a high proportion of midbrain dopaminergic neurons (manuscript in resubmission) (Figure 1B,C). These results were supported by qRT-PCR analysis showing a down-regulation of the pluripotency marker OCT4 and an up-regulation of the midbrain dopaminergic markers EN1, LMX1A, and GIRK2 (Supplementary Figures S1A–D). Calcium-imaging recordings indicated that the neurons were functionally matured as they displayed spontaneous activity and could be depolarized by potassium chloride (data not shown). This finding was further supported by immunocytochemistry showing a significant expression of the presynaptic marker synaptophysin in polarized neurons (Figure 1D).

Using the reported differentiation protocol, *PARK2* KO and isogenic control neurons from three independent differentiations were collected and subjected to mitochondrial enrichment prior to the proteomic analysis (Figure 1E), leading to the identification of a total of 5747 proteins (Figure 1F). Data are available via ProteomeXchange with identifier PXD008894. Based on the proteins identified with high confidence (more than or equal to two unique peptides), our analysis quantified levels of 945 out of 1564 mitochondrial proteins (60%) listed in the Gene Ontology (GO) Database (GO term: mitochondrion, organism: *Homo sapiens*, type: protein), giving us a comprehensive description of mitochondrial protein changes in human neurons in response to parkin dysfunction (Figure 1F,G). The average abundances of mitochondrial proteins were identical in the six samples, indicating that the enrichment procedure had resulted in similar amounts of mitochondria in each sample (Figure 1H). The average abundances of the neuronal markers TUJ1, MAP2, and synaptophysin were similar in control and *PARK2* KO samples (Figure 1I). Levels of 1064 proteins were significantly altered in the *PARK2* KO neurons, and 119 of these were connected to mitochondria (Figure 1F,G and Supplementary Tables S1A,B). In addition, we quantified levels of 4585 phosphorylated peptides of which 296 were mitochondrial. However, none of these were significantly altered, indicating that parkin dysfunction did not consistently change the mitochondrial phospho-proteome in this model (Supplementary Table S1C).

### Pathway Analysis of Proteomic Changes Reveals Disturbances in Cell Cycle Regulation, Oxidative Stress, and Energy Metabolism in *PARK2* KO Neurons

**FIGURE 2:**
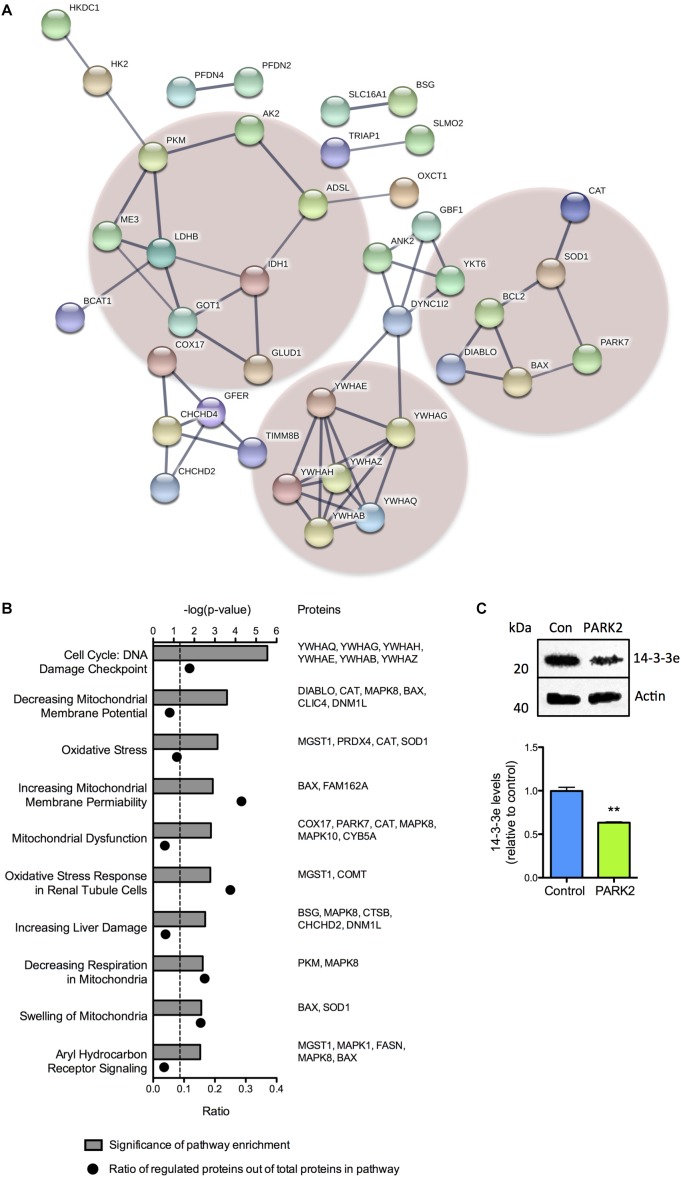
Cluster and pathway analysis of proteomic changes in *PARK2* KO neurons. **(A)** STRING analysis on the 119 significantly dysregulated mitochondrial proteins identified by the proteomic analysis, revealed three clusters of proteins related to lactate–pyruvate metabolism (left cluster), oxidative stress (right cluster), and cell cycle regulation and cell death (lower cluster), respectively. Only connected nodes are shown. The line thickness indicates the strength of the supporting data. **(B)** Top toxicity-related pathways identified by ingenuity pathway analysis (IPA) of the significantly dysregulated mitochondrial proteins. Bar graphs indicate the enrichment level of each pathway shown as –log(*P*-value). The corresponding dots indicate the ratio of identified proteins out of the total number of proteins in the pathway. **(C)** Western blotting confirming a significant decrease in the level of 14-3-3𝜀 in *PARK2* KO neurons. Protein expression levels were normalized to β-actin and shown relative to control neurons. Mean ± SEM, *n* = 3 technical replicates, data from three independent differentiations. ^∗∗^*P* < 0.01, Student’s *T*-test.

A large proportion of the differentially regulated mitochondrial proteins were related to oxidative stress defense, energy metabolism, and cell cycle regulation. This was demonstrated by STRING analysis, which revealed a cluster of 14-3-3 (YWHA-) proteins involved in cell cycle regulation and apoptosis signaling and two clusters containing proteins involved in defense against oxidative stress and energy metabolism, respectively (Figure 2A). Pathway analysis, based on differences in protein profiles, revealed that the central toxicity-related pathways included cell cycle check points for DNA damage and processes related to mitochondrial dysfunction including oxidative stress, decreasing respiration, and mitochondrial swelling among others (Figure 2B). The decreased level of 14-3-3𝜀 (YWHAE) was confirmed by Western blotting (Figure 2C), as were the lower level of BAX although with a large degree of variation in total BAX expression between differentiations (Supplementary Figure S2A).

**FIGURE 3:**
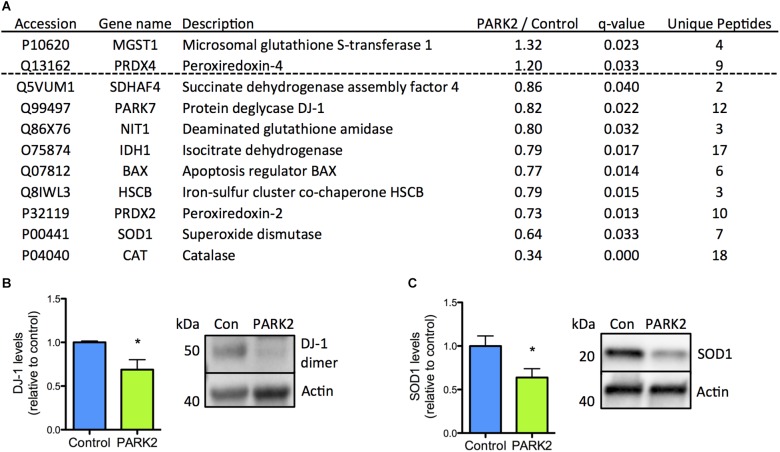
Confirmation of down-regulation of oxidative stress defense proteins identified by the proteomic analysis. **(A)** Table listing the proteins related to oxidative stress defense identified by the mitochondrial proteomic analysis with the ratio of protein levels in *PARK2* KO neurons compared to controls; *q*-value (FDR-adjusted *P*-value) and number of unique peptides, *n* = 3 independent differentiations. **(B,C)** Western blotting confirmed a significant decrease in levels of **(B)** DJ-1 and **(C)** superoxide dismutase 1 (SOD1) in *PARK2* KO neurons. Expression levels were normalized to β-actin and shown relative to control neurons. Mean ± SEM, *n* = 6 technical replicates, data from three independent differentiations. ^∗^*P* < 0.05, Student’s *T*-test.

The reduced levels of antioxidant enzymes in *PARK2* KO neurons were surprising, but could be confirmed, as the significant down-regulation of the PD-related protein DJ-1 as well as SOD1 was documented by Western blotting (Figure 3A–C). In addition we have earlier confirmed that significantly decreased levels of catalase are present in *PARK2* KO neurons, overall indicating that impairment in the defense against oxidative stress is apparent (manuscript in resubmission).

### Perturbations in Mitochondrial Area and Morphology in *PARK2* KO Neurons

**FIGURE 4:**
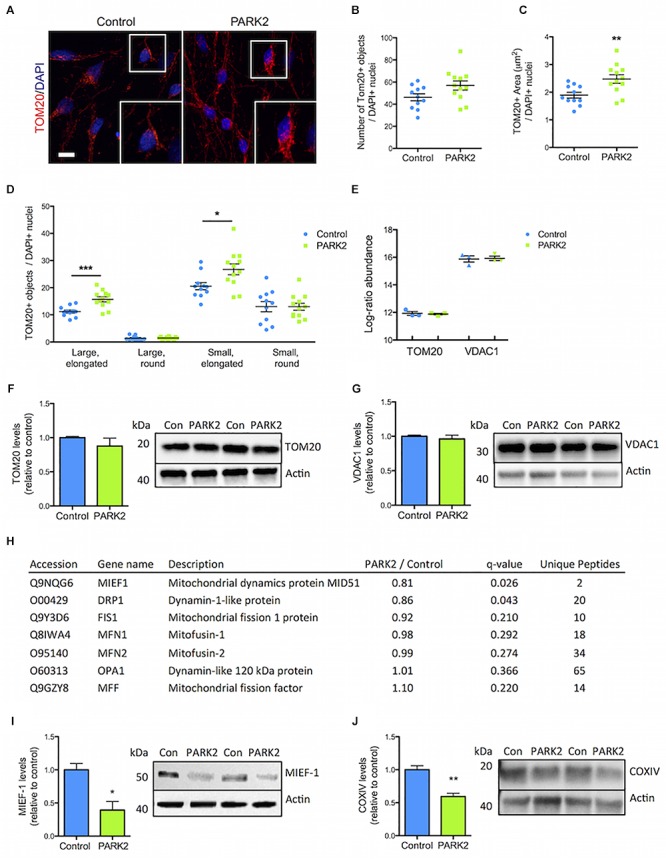
Perturbed mitochondrial morphology in *PARK2* KO neurons. **(A)** TOM20 (red) immunofluorescence staining and **(B,C)** quantification in control and *PARK2* KO neurons showed **(B)** unchanged numbers of TOM20+ objects and **(C)** significantly increased area of TOM20 staining when normalized to number of DAPI+ nuclei (blue). **(D)** The number of elongated mitochondria was significantly increased in *PARK2* KO cells, whereas the number of round mitochondria was unchanged, when separating TOM20+ objects depending on size (small vs. large) and shape (round vs. elongated). Scale bar = 10 μm. Mean ± SEM, *n* = 11–12 technical replicates, data from four independent differentiations. **(E)** The average proteomics-identified abundance (log ratio) of TOM20 and VDAC1 was similar between control and *PARK2* KO neurons. Mean ± SEM, *n* = 3 independent differentiations. **(F,G)** Western blotting showed no changes in **(F)** TOM20 and **(G)** VDAC1 levels. Protein expression levels were normalized to β-actin and shown relative to control neurons. Mean ± SEM, *n* = 4–10 technical replicates, data from three independent differentiations. **(H)** Table listing the proteins related to mitochondrial dynamics identified by the mitochondrial proteomic analysis with the ratio of protein levels in *PARK2* KO neurons compared to controls; *q*-value (FDR-adjusted *P*-value) and number of unique peptides, *n* = 3 independent differentiations. **(I,J)** Western blotting showed a significant decrease in the levels of **(I)** MIEF1 and **(J)** COXIV in *PARK2* KO neurons. Protein expression levels were normalized to β-actin and shown relative to control neurons. Mean ± SEM, *n* = 3–4 technical replicates, data from three independent differentiations. ^∗^*P* < 0.05, ^∗∗^*P* < 0.01, ^∗∗∗^*P* < 0.005, Student’s *T*-test.

The *PARK2* KO did not significantly affect the total number of mitochondria as measured by TOM20 immunofluorescence staining (Figure 4A,B). However, the *PARK2* KO neurons contained a significantly increased area of TOM20 immunoreactivity, indicating an overall larger mitochondrial area per cell (Figure 4A,C). To examine the mitochondrial morphology, we divided the mitochondria into four categories based on shape and size (Supplementary Figure S4). This demonstrated a significant increase in the numbers of elongated mitochondria (both large and small) in the *PARK2* KO cells, whereas numbers of round mitochondria were unchanged (Figure 4D). The proteomic data revealed identical levels of TOM20 (*PARK2* KO/control ratio = 0.99) and the mitochondrial marker, VDAC1 (*PARK2* KO/control ratio = 1.06) between the lines (Figure 4E). Western blotting for TOM20 and VDAC1 levels confirmed no difference between the lines (Figure 4F,G). This supported that the observed changes might be related to mitochondrial morphology rather than changes in numbers. Interestingly, levels of the mitochondrial dynamics protein MIEF1 and the dynamin-1-like protein (Drp1) were significantly decreased as shown by the proteomic analysis whereas levels of other key mitochondrial fission-fusion proteins were unaffected (Figure 4H). By Western blotting the decrease in the MIEF1 level was validated (Figure 4I), but no change in Drp1 levels was detected (Supplementary Figure S2B).

### PARK2 KO Neurons Are Deficient in Glycolysis and Lactate Metabolism

The proteomic analysis quantified large numbers of electron transport chain (ETC) proteins. Interestingly, cytochrome c oxidase (COX) IV levels were, as measured by Western blotting, significantly reduced in *PARK2* KO neurons (Figure 4J), which was in agreement with the proteomic data showing decreased levels of 3 complex IV-related proteins [COX copper chaperone (COX17), COX assembly factor 6 and 7 (COA6/7)] out of 21 identified (Supplementary Table S2). Forty-one complex I subunits or related proteins as well as 12 complex III proteins were identified (Supplementary Tables S3, S4), but none of these were significantly affected, indicating that the changes were specific for complex IV.

**FIGURE 5:**
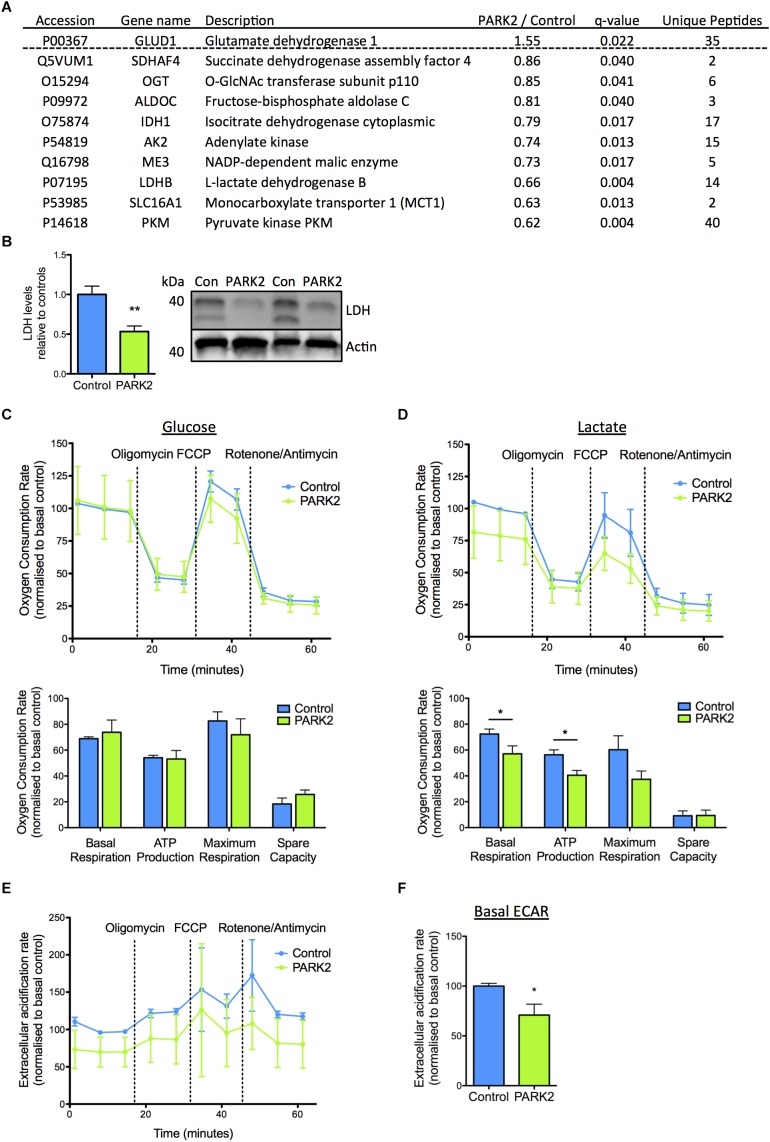
Functional changes in lactate metabolism in *PARK2* KO neurons support protein changes revealed by proteomic analysis. **(A)** Table listing the mitochondrial proteins related to lactate–pyruvate metabolism identified by the proteomic analysis with the ratio of protein levels in *PARK2* KO neurons compared to controls; *q*-value (FDR-adjusted *P*-value) and number of unique peptides, *n* = 3 independent differentiations. **(B)** Western blotting confirmed a significant decrease in levels of lactate dehydrogenase (LDH) in *PARK2* KO neurons. Expression levels were normalized to β-actin and shown relative to control neurons. Mean ± SEM, *n* = 6 technical replicates, data from three independent differentiations. **(C–F)** Oxygen consumption rates (OCRs) and extracellular acidification rates (ECARs) of *PARK2* KO and control neurons normalized to protein content illustrating basal respiration, the respiration coupled to mitochondrial ATP production after oligomycin treatment, maximal respiration after FCCP treatment, and non-mitochondrial respiration after rotenone/antimycin treatment. The spare respiratory activity is defined as the difference between the baseline and FCCP-induced maximal respiration. **(C)** *PARK2* KO neurons had similar OCR when incubated in medium with 2.5 mM glucose and **(D)** significantly decreased basal respiration and ATP production in medium with 2.5 mM lactate. **(E,F)** Basal ECAR was significantly decreased in *PARK2* KO neurons. Mean ± SEM, *n* = 3–9 technical replicates, data from three independent differentiations. ^∗^*P* < 0.05, ^∗∗^*P* < 0.01, Student’s *T*-test.

In addition, a number of the mitochondrial proteins dysregulated in *PARK2* KO neurons were of importance for energy metabolism and the pathway analysis pointed to a decrease in mitochondrial respiration. The proteins related to energy metabolism were almost exclusively down-regulated with LDH B, pyruvate kinase PKM (PKM), and monocarboxylate transporter 1 displaying the most significantly decreased levels (Figure 5A). Significantly decreased levels of LDH were confirmed by Western blotting (Figure 5B). These observations correlated with a significantly decreased ability of *PARK2* KO neurons to respire using lactate as a substrate. This was demonstrated by measuring OCRs of control and *PARK2* KO neurons in medium with either glucose or lactate as the only energy source (Figure 5C,D). Both the basal OCR and ATP production was significantly reduced in *PARK2* KO neurons respiring on lactate, confirming a disturbance in lactate metabolism (Figure 5D and Supplementary Figure S3). With glucose no difference in OCR between control and *PARK2* KO neurons was observed despite the decreased COXIV levels (Figure 4J). However, the basal ECAR with glucose was significantly decreased, indicating an impairment of glycolysis in *PARK2* KO neurons (Figure 5E,F).

### Decreased Proliferation and Survival of *PARK2* KO Cells

**FIGURE 6:**
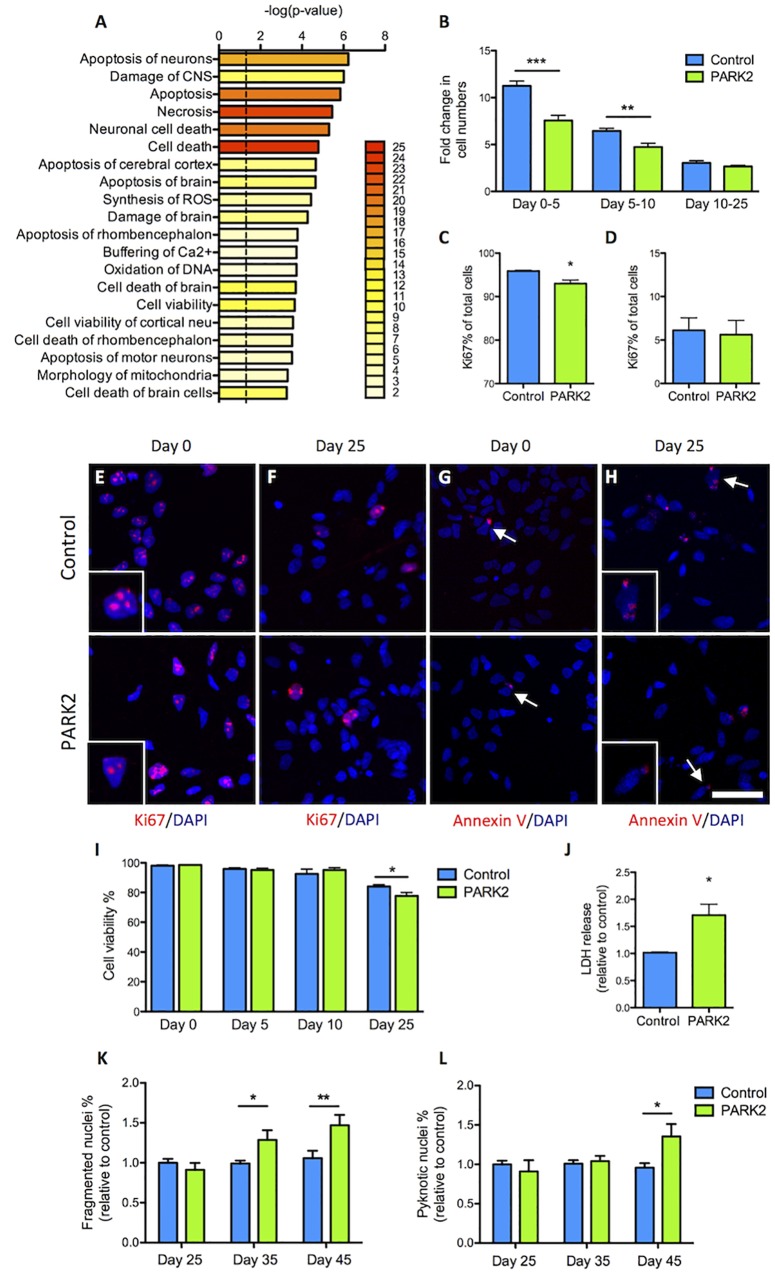
Cell proliferation and survival is impeded in *PARK2* KO neurons. **(A)** Pathway analysis on the differentially expressed proteins showing with bar graphs the enrichment score –log 10(*P*-value) and with color labeling the number of identified proteins belonging to each pathway. Only pathways with two or more proteins are shown. **(B)** Cell counts on differentiation days 0, 5, 10, and 25 from the NSC stage indicated that the fold change in cell numbers from days 0–5 and 5–10 was significantly lower in *PARK2* KO cells compared to isogenic controls, whereas from days 10 to 25 there was no such difference between the lines. Mean ± SEM, *n* = 5–10 independent differentiations. **(C,E)** Quantification of immunofluorescence staining for Ki67+ (red) cells at differentiation day 0 revealed significantly lower levels of proliferation in *PARK2* KO cultures. **(D,F)** At differentiation day 25, no difference in the relative content of Ki67+ cells was observed between *PARK2* KO and control neurons. Mean ± SEM, *N* = 2 independent differentiations. **(G,H)** Immunofluorescence staining for Annexin V (red) on control and *PARK2* KO cells on differentiation day 0 and day 25 identified very few apoptotic cells in both cell lines. Cell nuclei were stained with DAPI (blue). Scale bar = 50 μm. **(I)** Cell viability as measured by Trypan blue staining of cells in suspension at differentiation days 0, 5, 10, and 25 showed significantly decreased survival of *PARK2* KO neurons at day 25. Mean ± SEM, *N* = 4 independent differentiations. **(J)** LDH release to the medium was increased in *PARK2* KO cultures compared to isogenic controls at day 25. Data presented relative to average controls. Mean ± SEM, *n* = 3 independent differentiations. **(K,L)** Analysis of nuclear morphologies at differentiation days 25, 35, and 45 indicating the percentage of fragmented and pyknotic nuclei as a proportion of total cells per field of view. Mean ± SEM, *N* = 15–36 fields of view, data from two independent differentiations. ^∗^*P* < 0.05, ^∗∗^*P* < 0.01, ^∗∗∗^*P* < 0.001, Student’s *T*-test.

Pathway analysis based on the 119 dysregulated proteins indicated that cell cycle regulation, cell viability, and neuronal cell death were affected in *PARK2* KO neurons (Figure 6A). We therefore sought to first determine proliferation rates during the differentiation. At the early time points similar numbers of cells gave rise to much fewer *PARK2* KO cells 5 days later (Figure 6B). This correlated with a significantly lower percentage of Ki67+ proliferative cells in the *PARK2* KO cultures at day 0 (control: 95.9 ± 0.2%, *PARK2*: 93.0 ± 0.8%, *P* < 0.01) (Figure 6C,E) while similar numbers were found at day 25 from the NSC stage (control: 6.1 ± 1.4%, *PARK2*: 5.6 ± 1.6%) (Figure 6D,F). To investigate cell viability, we examined the number of Annexin V+ apoptotic cells, which was very low for both cell lines at days 0 and 25 (Figure 6G,H). This indicated low levels of apoptotic cell death in general. However, Trypan blue staining of cells in suspension after accutase treatment revealed a significantly lower viability of the *PARK2* KO neurons at day 25 (control: 84.1 ± 1.2%, *PARK2*: 77.7 ± 2.3%, *P* < 0.05) (Figure 6I), which was confirmed by a significantly higher LDH release from the *PARK2* KO cultures at day 25, indicating increased necrosis (Figure 6J). Analysis of the nuclear morphologies at days 25, 35, and 45 revealed significantly increased percentages of fragmented and pyknotic nuclei for *PARK2* KO neurons relative to controls at the later time points, indicating higher levels of cell death over time (Figure 6K,L). In conclusion, both cell proliferation and neuronal cell survival was altered for *PARK2* KO cells.

## Discussion

The importance of mitochondrial dysfunction in sporadic and certain forms of familial PD is well established, but the specific mechanisms and pathways are not well understood (Abou-Sleiman et al., 2006). Human iPSC-derived neurons with *PARK2* KO allow for the study of chronic mitochondrial dysfunction in PD without toxic insults or other stressors (Imaizumi et al., 2012; Jiang et al., 2012; Chung et al., 2016). By applying isogenic iPSC-derived neurons and large-scale mass spectrometry-based proteomics, we have been able to investigate the effect of parkin dysfunction on large numbers of proteins. Using this approach we have identified 60% of the known mitochondrial proteins of which 119 were significantly dysregulated in *PARK2* KO neurons.

Several proteins of importance for the cellular defense against oxidative stress were reduced in *PARK2* KO neurons, rendering them more susceptible to oxidative damage. An earlier study on patient fibroblasts with *PARK2* mutation has similarly reported decreased levels and/or activity of catalase and superoxide dismutase (SOD) 2 (Pacelli et al., 2011). Palacino et al. (2004) identified 15 proteins, with altered levels in *PARK2* KO mice, of which 4 involved in protection against oxidative stress displayed decreased levels. One of these, peroxiredoxin-2, was significantly reduced in the *PARK2* KO neurons (Palacino et al., 2004). As the mitochondrial dysfunction caused by parkin deficiency can independently increase oxidative stress levels, a compensatory increase in proteins such as DJ-1, catalase, and SOD1/2 could have been expected (Abou-Sleiman et al., 2006). These results, however, indicate that *PARK2* KO has direct negative impact on antioxidant enzyme levels.

Earlier proteomic studies applying mitochondrial enrichment to toxin-based animal and cell models have shown limited commonality in the identified regulated proteins (Aroso et al., 2016). However, two proteins that have been consistently changed across different studies, including in post-mortem PD patient brain tissue, are PKM and 14-3-3 epsilon, which were among the most significantly down-regulated proteins in our *PARK2* KO neurons (Periquet et al., 2005; Jin et al., 2006; Aroso et al., 2016). PKM is the last enzyme in the glycolysis pathway and corresponding with this, decreased ECAR, an indirect measure of glycolysis, was present in *PARK2* KO neurons metabolizing glucose. Periquet et al. (2005) in their study of proteomic changes in *PARK2* KO mice found decreased levels of both PKM, LDH, fructose-bisphosphate aldolase C (ALDOC), agreeing with our results, and also of pyruvate dehydrogenase, which converts pyruvate to acetyl CoA. Out of the 15 dysregulated proteins identified in Palacino et al. (2004), pyruvate dehydrogenase was the one showing the highest reduction in *PARK2* KO mice. A proteomic study on PINK1 KO rat brain similarly documented changes in levels of glycolysis-related proteins, including LDH, which were increased, and PKM, which was reduced (Villeneuve et al., 2016). Overall this indicates that changes in glycolytic flux could be generally present in PD. A study on human cancer cell lines identified PKM2, the PKM splice form present in cancer cells, as a parkin substrate and demonstrated that parkin KO caused increased activity of PKM2 and glycolysis in general (Liu et al., 2016). Although these results appear conflicting they do point to a link between parkin dysfunction and glycolysis.

Functionally, *PARK2* KO neurons incubated without glucose supplementation and having only lactate as a substrate exhibited significantly decreased basal respiration and ATP production, confirming that in addition to glycolysis, lactate metabolism is also perturbed in *PARK2* KO neurons.

Accumulating evidence indicates that astrocyte-derived lactate is an important energy source for neurons and neuroprotective in pathological situations (Wyss et al., 2011; Jourdain et al., 2016). However, in mice an age-dependent increase in brain lactate levels happens as a result of mitochondrial dysfunction promoting pyruvate to lactate conversion (Ross et al., 2010; Quistorff and Grunnet, 2011). In our *PARK2* KO neurons, the observed reduction in LDH and monocarboxylase transporter 1 may explain the decreased capacity of the cells to utilize lactate (Supplementary Figure S3). Combined with a reduction in glycolytic capacity, such metabolic changes may lead to harmful energy deficits.

A significant decrease in complex IV levels was also documented in the *PARK2* KO neurons. As important components of complex IV are encoded by mitochondrial DNA (mtDNA), mitochondrial dysfunction and oxidative stress causing damage to mtDNA could potentially explain this (Ross et al., 2010). Earlier studies have linked mtDNA damage and complex IV dysfunction (Bender et al., 2006; Kraytsberg et al., 2006). It remains to be determined whether higher levels of mtDNA damage are present in our *PARK2* KO neurons; however, a recent study has documented this in *PARK2* KO neurons from mice (Pinto et al., 2018).

Despite the decrease in complex IV levels, no effect on OCR was observed when glucose was present in the medium. However, the lower complex IV level could perhaps be a rate-limiting factor when lactate is the substrate. Palacino et al. (2004) found a reduction of proteins from both complexes I and IV, correlating with a decreased respiratory capacity of mitochondria in *PARK2* KO mice. Other studies, addressing OCR in the context of parkin dysfunction, have examined fibroblasts from *PARK2* PD patients and reported contradictory results of decreased or increased basal and maximum OCR (Pacelli et al., 2011; Zanellati et al., 2015). The discrepancy between these studies and also the present study remains unclear, but perhaps differences in energy metabolism between fibroblasts and neurons play a role.

The proteomic analysis revealed that known parkin substrates such as TOM20, VDAC1, Drp1, and MFN1/2 are not accumulating in this model. Earlier studies of proteomic changes in *PARK2* KO mice show similar results (Palacino et al., 2004; Periquet et al., 2005).

The TOM20 IF staining for mitochondria revealed increased overall area of immunoreactivity and enhanced numbers of elongated mitochondria in the *PARK2* KO neurons, whereas the levels of TOM20 and VDAC1 protein were unchanged. This indicates that the total mitochondrial content is not affected in the *PARK2* KO neurons, but that mitochondrial morphology is perturbed in the direction of larger, more elongated mitochondria (Zanellati et al., 2015; Pinto et al., 2018). This corroborates earlier results indicating increased swelling of mitochondria in *PARK2* KO neurons (Shaltouki et al., 2015). This may be explained by the role of parkin in promoting mitochondrial fission through Drp1 and is supported by reports of decreased fission and mitochondrial swelling observed for other parkin dysfunction models (Poole et al., 2008; Buhlman et al., 2014; Chung et al., 2016). However, others have reported increased mitochondrial fragmentation as a result of acute down-regulation of parkin (Lutz et al., 2009). The mitochondrial enlargement observed in the present model may reflect a compensatory response, given that mitochondrial swelling, as a result of altered fission-fusion processes, has been reported to occur to maintain oxidative phosphorylation and increase respiratory activity under stress (MacVicar and Lane, 2014; Mishra et al., 2014). Interestingly, Drp1 and MIEF1, which promotes mitochondrial fusion and inhibits fission, were found to be significantly decreased in *PARK2* KO neurons by proteomic analysis, which for MIEF1 was confirmed by Western blotting. Although no significant phosphorylation changes were detected in the *PARK2* KO neurons, MIEF1 was among the proteins with high levels of phosphorylation on several known phospho-sites and could potentially be involved in the observed morphological changes (Zhao et al., 2011).

The above-mentioned changes in oxidative stress defense and energy metabolism could be a cause of the documented impairment of proliferation and survival of *PARK2* KO cells. Although *PARK2* mutations cause early-onset PD, they are not known to affect neurodevelopment and proliferation of NSCs *in vivo*. The increased oxidative stress related to *in vitro* conditions could explain this discrepancy as resulting DNA damage could negatively impact proliferation (Jagannathan et al., 2016). In relation to this, six out of seven of the 14-3-3 proteins, which are known to regulate the cell cycle and promote cell survival by inhibiting apoptosis (Xing et al., 2000), were decreased in our *PARK2* KO neurons, which for 14-3-3𝜀 was confirmed by Western blotting. 14-3-3 proteins can interact with both α-synuclein and parkin and their overexpression has neuroprotective effects in PD cell and animal models (Xing et al., 2000; Yacoubian et al., 2010). As earlier mentioned, 14-3-3𝜀 is one out of two proteins consistently found decreased in proteomics studies of PD (Aroso et al., 2016). Interestingly, the decrease in survival was not evident until the final stages of the differentiation where MAP2+ neurons were present, indicating perhaps an increased vulnerability of the post-mitotic neurons.

In conclusion, we have applied advanced mass spectrometry-based proteomics to obtain a comprehensive characterization of mitochondrial protein changes in response to *PARK2* KO in isogenic human iPSC-derived neurons with perturbed mitochondrial morphology. This resulted in the identification of dysfunctions in oxidative stress defense, glycolysis, and lactate–pyruvate metabolism as well as impaired cell survival, adding valuable new insight into the role of parkin dysfunction in PD pathogenesis.

## Author Contributions

HB, PJ, ML, and MM designed the research. HB, PJ, MR, SS, JO, MH, LR, CW, and CA performed the research. LR, LB, HW, and RW-M contributed new reagents or analytic tools. HB, PJ, MR, SS, JO, BR, MH, and CA analyzed the data. HB, LB, BR, and MM wrote the manuscript.

## Conflict of Interest Statement

The authors declare that the research was conducted in the absence of any commercial or financial relationships that could be construed as a potential conflict of interest.
