# Supplementary material for: PARK2 Mutation Causes Metabolic Disturbances and Impaired Survival of Human iPSC-Derived Neurons
Source: Front Cell Neurosci. 2019 Jul 5;13:297. doi: 10.3389/fncel.2019.00297 (PMC6624735; doi:10.3389/fncel.2019.00297)
Supplement: Supplementary file 2 [file Data_Sheet_1.pdf]

Supplemental Information

Fig. S1: Midbrain dopaminergic differentiation of *PARK2* KO and isogenic control neuronal cultures. (A-D) qRT-PCR analysis for mRNA levels, normalised to glyceraldehyde 3-phosphate dehydrogenase (GAPDH) show (A) down-regulation of the pluripotency marker OCT4 and upregulation of the midbrain dopaminergic markers (B) homeobox protein engrailed-1 (EN1), (C) LIM homeobox transcription factor 1 α (LMX1A), and (D) G protein-activated inward rectifier potassium channel 2 (GIRK2). Mean ± SEM, n = 4, data from 2 independent differentiations, $^{**}P<0.01$, $^{***}P<0.005$, one-way ANOVA with Tukey's Multiple Comparison test.

Fig. S2: Western blotting for BAX and Drp1. (A) Western blotting using samples from 3 independent differentiations showing large variation in expression of BAX with a marked decreased in *PARK2* KO neurons compared to control in one differentiation. (B) Western blotting showed similar levels of Drp1 in control and *PARK2* KO neurons. Expression levels were normalized to β-actin and shown relative to control neurons. Mean ± SEM, n = 3 independent differentiations.

Fig. S3: Pathway analysis identified changes in glycolysis and lactate-pyruvate metabolism in *PARK2* KO neurons. Simplified schematic showing the glycolysis and pyruvate-lactate pathways with the down-regulated proteins in blue: fructose-bisphosphate aldolase C (ALDOC), pyruvate kinase PKM (PKM), lactate dehydrogenase B (LDHB) and monocarboxylase transporter 1 (MCT1).

Fig. S4: Mitochondrial morphology analysis. (A, C) Immunofluorescence staining of control and *PARK2* KO neurons on differentiation day 25 for mitochondrial import receptor subunit TOM20 (red) and nuclei (DAPI, blue). (B, D) Binary images of all TOM20+ mitochondria between 0.15-3.50 μm^2 in size. (E, G) Large, elongated mitochondria between 0.71-3.50 μm^2 with circularity of 0.01-0.50. (F, H) Large, round mitochondria between 0.71-3.50 μm^2 with circularity of 0.50-1.00. (I, K) Small, elongated mitochondria between 0.15-0.70 μm^2 with circularity of 0.01-0.50. (J, L) Small, round mitochondria between 0.15-0.70 μm^2 with circularity of 0.50-1.00.

Table S1: Table listing (A) all the significantly regulated proteins in the proteomic analysis, (B) all the significantly regulated mitochondrial proteins in the proteomic analysis, and (C) all the mitochondrial proteins with phosphorylation changes in the proteomic analysis.

Table S2: Table listing the electron transport chain complex IV proteins identified by the proteomic analysis with the ratio of protein levels in *PARK2* KO neurons compared to controls, p-value based on Student's T-test and number of unique peptides, n = 3 independent differentiations.

Table S3: Table listing the electron transport chain complex I proteins identified by the proteomic analysis with the ratio of protein levels in *PARK2* KO neurons compared to controls, p-value based on Student's T-test and number of unique peptides, n = 3 independent differentiations.

Table S4: Table listing the electron transport chain complex III proteins identified by the proteomic analysis with the ratio of protein levels in *PARK2* KO neurons compared to

controls, p-value based on Student's T-test and number of unique peptides, n = 3 independent differentiations.

Fig. S1

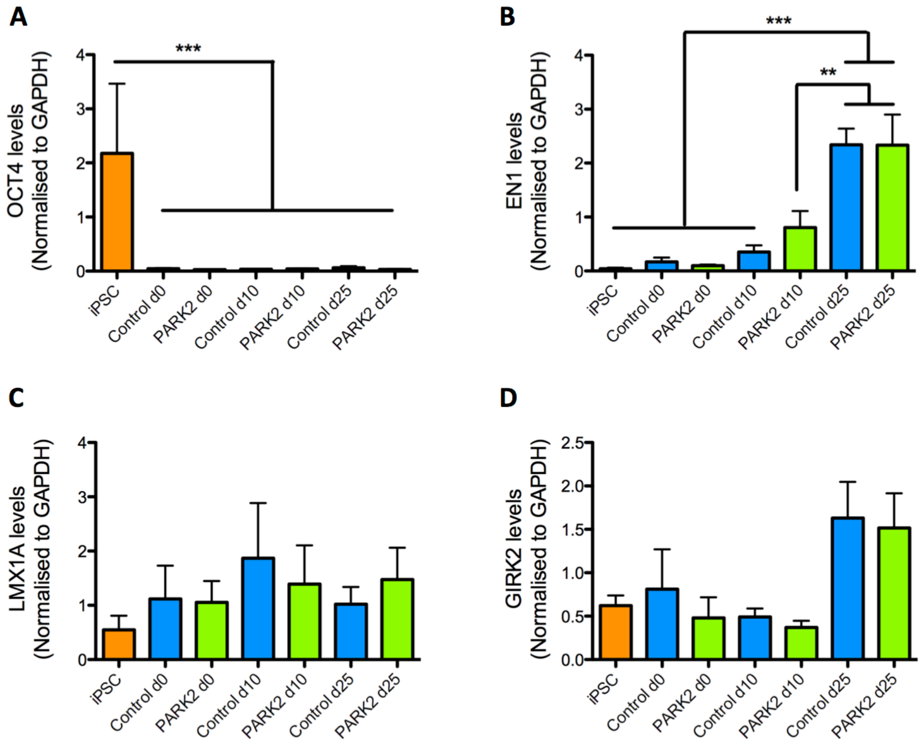
A
OCT4 levels
(Normalised to GAPDH)
***
0
1
2
3
4
iPSC
Control d0
PARK2 d0
Control d10
PARK2 d10
Control d25
PARK2 d25
B
EN1 levels
(Normalised to GAPDH)
***
**
0
1
2
3
4
iPSC
Control d0
PARK2 d0
Control d10
PARK2 d10
Control d25
PARK2 d25
C
LMX1A levels
(Normalised to GAPDH)
0
1
2
3
4
iPSC
Control d0
PARK2 d0
Control d10
PARK2 d10
Control d25
PARK2 d25
D
GIRK2 levels
(Normalised to GAPDH)
0.0
0.5
1.0
1.5
2.0
2.5
iPSC
Control d0
PARK2 d0
Control d10
PARK2 d10
Control d25
PARK2 d25

Fig. S2

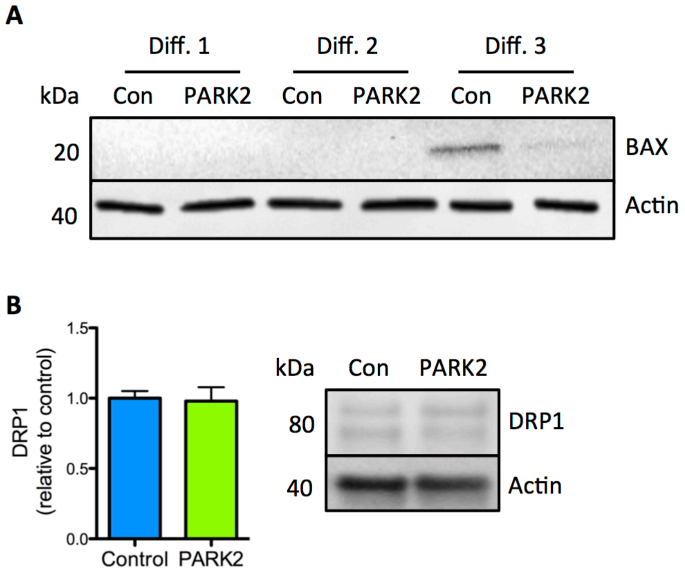
A
Diff. 1
Diff. 2
Diff. 3
kDa
Con
PARK2
Con
PARK2
Con
PARK2
20
40
BAX
Actin
B
DRP1 (relative to control)
1.5
1.0
0.5
0.0
Control
PARK2
kDa
Con
PARK2
80
40
DRP1
Actin

1 **Fig. S3**

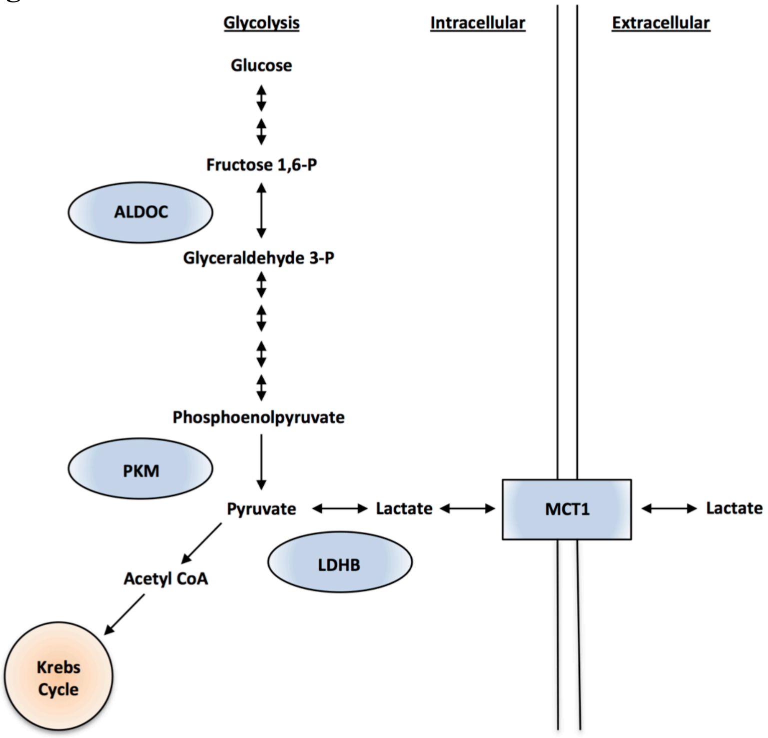
Glycolysis
Intracellular
Extracellular
Glucose
Fructose 1,6-P
ALDOC
Glyceraldehyde 3-P
Phosphoenolpyruvate
PKM
Pyruvate
Lactate
MCT1
Lactate
Acetyl CoA
LDHB
Krebs Cycle

Fig. S4

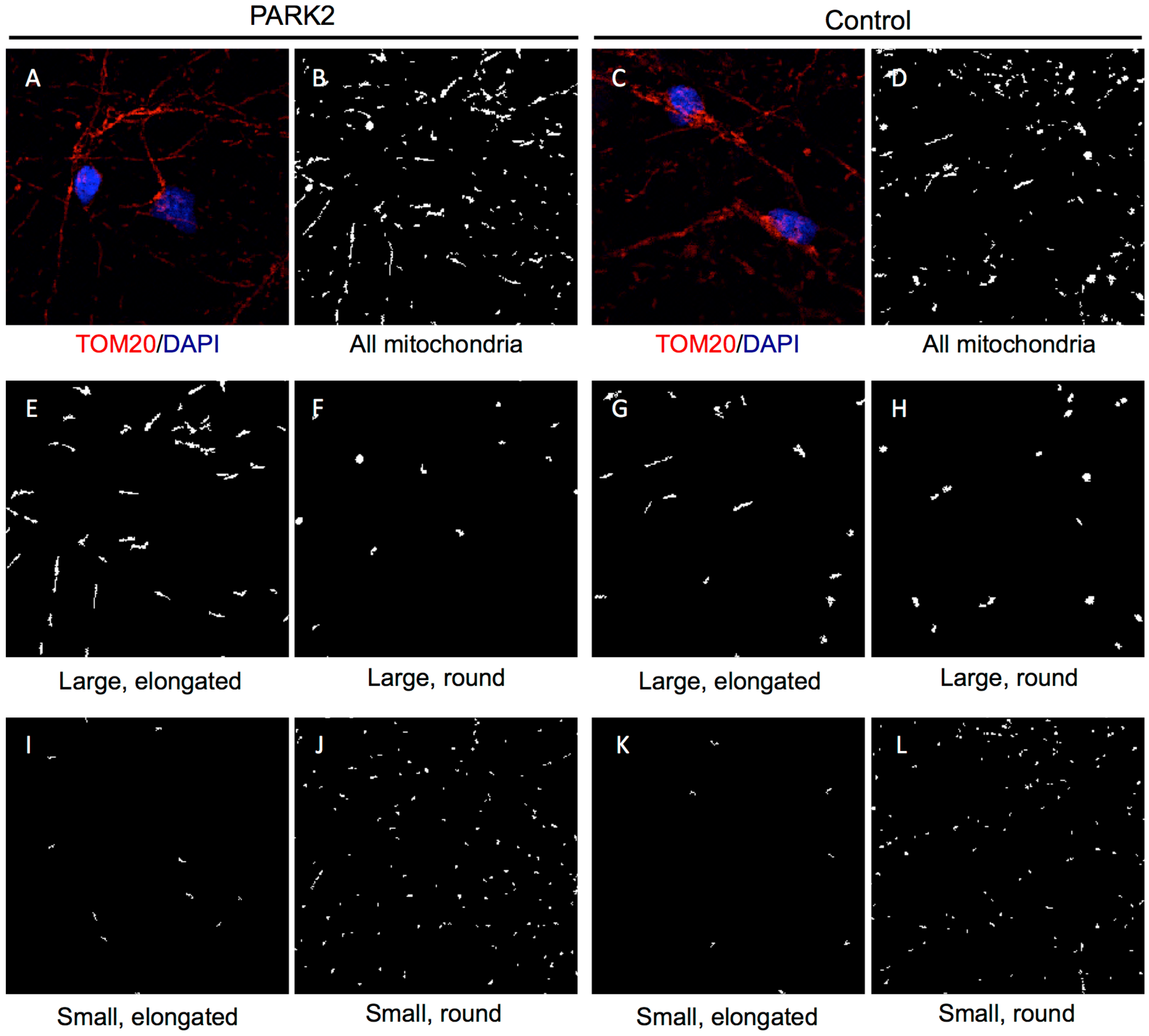
PARK2
Control
A
B
C
D
TOM20/DAPI
All mitochondria
TOM20/DAPI
All mitochondria
E
F
G
H
Large, elongated
Large, round
Large, elongated
Large, round
I
J
K
L
Small, elongated
Small, round
Small, elongated
Small, round

Table S2

Complex IV proteins

Accession	Gene name	Description	PARK2 / Control	P-value
Q9GZY4	COA1	Cytochrome c oxidase assembly factor 1 homolog	0,858	0,115
Q9Y2R0	COA3	Cytochrome c oxidase assembly factor 3 homolog	0,97	0,177
**Q86WW8**	**COA5**	**Cytochrome c oxidase assembly factor 5**	**0,76**	**0,042**
**Q5JTJ3**	**COA6**	**Cytochrome c oxidase assembly factor 6 homolog**	**0,76**	**0,015**
**Q96BR5**	**COA7**	**Cytochrome c oxidase assembly factor 7**	**0,767**	**0,012**
P00395	COX1	Cytochrome c oxidase subunit 1	1,342	0,078
Q96I36	COX14	Cytochrome c oxidase assembly protein COX14	0,901	0,113
Q7KZN9	COX15	Cytochrome c oxidase assembly protein COX15	1,177	0,165
**Q14061**	**COX17**	**Cytochrome c oxidase copper chaperone**	**0,67**	**0,013**
Q8N8Q8	COX18	Cytochrome c oxidase assembly protein COX18	1,126	0,335
P00403	COX2	Cytochrome c oxidase subunit 2	0,972	0,429
Q5RI15	COX20	Cytochrome c oxidase assembly protein COX20	1,122	0,181
P13073	COX4I1	Cytochrome c oxidase subunit 4 isoform 1	0,977	0,152
P20674	COX5A	Cytochrome c oxidase subunit 5A	1,119	0,293
P10606	COX5B	Cytochrome c oxidase subunit 5B	0,925	0,160
P12074	COX6A1	Cytochrome c oxidase subunit 6A1	1,104	0,219
P14854	COX6B1	Cytochrome c oxidase subunit 6B1	0,983	0,217
P09669	COX6C	Cytochrome c oxidase subunit 6C	1,059	0,194
P14406	COX7A2	Cytochrome c oxidase subunit 7A2	1,000	0,358
O14548	COX7A2L	Cytochrome c oxidase subunit 7A-related protein	0,912	0,241
P24311	COX7B	Cytochrome c oxidase subunit 7B	0,912	0,241
P15954	COX7C	Cytochrome c oxidase subunit 7C	1,029	0,274
P99999	CYCS	Cytochrome c	0,844	0,321
P53701	HCCS	Cytochrome c-type heme lyase	0,996	0,361
O00483	NDUFA4	Cytochrome c oxidase subunit NDUFA4	0,952	0,210

Table S3

Complex I proteins

Accession	Gene name	Description	PARK2 / Control	P-value
O95299	NDUFA10	NADH dehydrogenase 1 alpha subcomplex subunit 10	1,094	0,397
Q86Y39	NDUFA11	NADH dehydrogenase 1 alpha subcomplex subunit 11	1,152	0,239
Q9UI09	NDUFA12	NADH dehydrogenase 1 alpha subcomplex subunit 12	1,127	0,341
Q9P0J0	NDUFA13	NADH dehydrogenase 1 alpha subcomplex subunit 13	0,968	0,264
O43678	NDUFA2	NADH dehydrogenase 1 alpha subcomplex subunit 2	1,05	0,362
O95167	NDUFA3	NADH dehydrogenase 1 alpha subcomplex subunit 3	1,336	0,088
Q16718	NDUFA5	NADH dehydrogenase 1 alpha subcomplex subunit 5	1,109	0,356
P56556	NDUFA6	NADH dehydrogenase 1 alpha subcomplex subunit 6	1,056	0,244
O95182	NDUFA7	NADH dehydrogenase 1 alpha subcomplex subunit 7	0,921	0,247
P51970	NDUFA8	NADH dehydrogenase 1 alpha subcomplex subunit 8	0,999	0,443
Q16795	NDUFA9	NADH dehydrogenase 1 alpha subcomplex subunit 9	1,076	0,436
O14561	NDUFAB1	Acyl carrier protein	1,067	0,208
Q9Y375	NDUFAF1	Complex I intermediate-associated protein 30	1,018	0,344
Q8N183	NDUFAF2	NADH dehydrogenase 1 alpha subcomplex assembly factor 2	1,024	0,436
Q9BU61	NDUFAF3	NADH dehydrogenase 1 alpha subcomplex assembly factor 3	0,94	0,444
Q9P032	NDUFAF4	NADH dehydrogenase 1 alpha subcomplex assembly factor 4	1,033	0,426
Q7L592	NDUFAF7	Protein arginine methyltransferase NDUFAF7	1,061	0,354
O75438	NDUFB1	NADH dehydrogenase 1 beta subcomplex subunit 1	1,02	0,317
O96000	NDUFB10	NADH dehydrogenase 1 beta subcomplex subunit 10	1,141	0,184
Q9NX14	NDUFB11	NADH dehydrogenase 1 beta subcomplex subunit 11	0,977	0,446
O95178	NDUFB2	NADH dehydrogenase 1 beta subcomplex subunit 2	0,958	0,421
O43676	NDUFB3	NADH dehydrogenase 1 beta subcomplex subunit 3	1,058	0,289
O95168	NDUFB4	NADH dehydrogenase 1 beta subcomplex subunit 4	0,982	0,273
O43674	NDUFB5	NADH dehydrogenase 1 beta subcomplex subunit 5	1,035	0,378
O95139	NDUFB6	NADH dehydrogenase 1 beta subcomplex subunit 6	1	0,433
P17568	NDUFB7	NADH dehydrogenase 1 beta subcomplex subunit 7	1,07	0,356
O95169	NDUFB8	NADH dehydrogenase 1 beta subcomplex subunit 8	1,086	0,340
Q9Y6M9	NDUFB9	NADH dehydrogenase 1 beta subcomplex subunit 9	0,989	0,266
O43677	NDUFC1	NADH dehydrogenase 1 subunit C1	0,961	0,286
O95298	NDUFC2	NADH dehydrogenase 1 subunit C2	0,941	0,292
P28331	NDUFS1	NADH-ubiquinone oxidoreductase 75 kDa subunit	1,121	0,341
O75306	NDUFS2	NADH dehydrogenase iron-sulfur protein 2	1,108	0,424
O75489	NDUFS3	NADH dehydrogenase iron-sulfur protein 3	1,094	0,378
O43181	NDUFS4	NADH dehydrogenase iron-sulfur protein 4	1,1	0,245
O43920	NDUFS5	NADH dehydrogenase iron-sulfur protein 5	1,022	0,293
O75380	NDUFS6	NADH dehydrogenase iron-sulfur protein 6,	1,018	0,293
O75251	NDUFS7	NADH dehydrogenase iron-sulfur protein 7	1,114	0,436
O00217	NDUFS8	NADH dehydrogenase iron-sulfur protein 8	1,052	0,421
P49821	NDUFV1	NADH dehydrogenase flavoprotein 1	1,097	0,373
P19404	NDUFV2	NADH dehydrogenase flavoprotein 2	0,973	0,394
P56181	NDUFV3	NADH dehydrogenase flavoprotein 3	1,117	0,132

Table S4

Complex III proteins

Accession	Gene name	Description	PARK2 / Control	P-value
P08574	CYC1	Cytochrome c1, heme protein,	1,117	0,245
Q9NVA1	UQCC1	Ubiquinol-cytochrome-c reductase complex assembly factor 1	0,998	0,211
Q9BRT2	UQCC2	Ubiquinol-cytochrome-c reductase complex assembly factor 2	1,04	0,278
Q6UW78	UQCC3	Ubiquinol-cytochrome-c reductase complex assembly factor 3	0,847	0,095
Q9UDW1	UQCR10	Cytochrome b-c1 complex subunit 9	1,094	0,384
O14957	UQCR11	Cytochrome b-c1 complex subunit 10	1,286	0,101
P14927	UQCRB	Cytochrome b-c1 complex subunit 7	1,117	0,419
P31930	UQCRC1	Cytochrome b-c1 complex subunit 1	1,175	0,311
P22695	UQCRC2	Cytochrome b-c1 complex subunit 2	0,981	0,307
P47985	UQCRFS1	Cytochrome b-c1 complex subunit Rieske	1,123	0,267
P07919	UQCRH	Cytochrome b-c1 complex subunit 6	0,925	0,163
O14949	UQCRQ	Cytochrome b-c1 complex subunit 8	1,119	0,247

Supplementary methods

Propagation of induced pluripotent stem cells (iPSCs)

IPSCs were propagated on Geltrex-coated plates (ThermoFisher) in iPSC medium, conditioned on mouse embryonic fibroblast feeder cells (MEFs, Amsbio) for 24 hrs. iPSC medium consisted of Dulbecco's Modified Eagle Medium (DMEM)/F12 (ThermoFisher) supplemented with 20% Knock-out Serum Replacement (KSR, ThermoFisher), 100 μM non-essential amino acids (NEAA, ThermoFisher), 4 mM GlutaMax-1 (ThermoFisher), 4 μM 2-Mercaptoethanol (ThermoFisher), 1% penicillin-streptomycin (pen-strep, ThermoFisher), and 4 ng/ml basic fibroblast growth factor (bFGF, R&D Systems). IPSCs were passaged 1:3-6 once a week, using 1 mg/ml collagenase IV (ThermoFisher) for 30 min and medium was changed every day. When thawing, freezing or passaging the iPSCs, ROCK inhibitor (Y27632, Stemgent) was added to promote survival.

Neural stem cell (NSC) propagation and differentiation

NSCs were propagated on Geltrex-coated plates in Neurobasal Medium (ThermoFisher) supplemented with NEAA, GlutaMax-I, B27 supplement (ThermoFisher), pen-strep, and bFGF. NSCs were passaged 1:3 when 80-90% confluent, by dissociating the cells for 5 min with accutase (ThermoFisher).

For differentiation, NSCs were seeded onto poly-L-ornithine- (Sigma) and laminin-coated (ThermoFisher) plates at a density of 50,000 cells/cm^2. DOPA Induction Medium (XCell Science) with addition of DOPA Induction Supplement A, B and C (XCell Science), and 200 ng/ml human recombinant sonic hedgehog (Peprotech) was added every other day for the first 9 days of differentiation. Cells were passaged using accutase for 5 min at day 5 and 10, counted using a hemocytometer and plated at a density of 50,000 cells/cm^2 unless otherwise specified. At day 10, the medium was changed to DOPA Maturation Medium (XCell Science) with DOPA Maturation Supplement A (XCell Science) until day 15 and DOPA Maturation Supplement B (XCell Science) hereafter. The resulting neurons were applied in assays from day 25 on unless otherwise stated.

Quantitative real time polymerase chain reaction (qRT-PCR)

For mRNA extraction, cells were harvested in Trizol lysis reagent (Life Technologies) and lysed by 30 min vortex. RNA purification was performed using the ENZA total RNA Kit I (VWR) according to manufacturer instructions. For reverse transcription from mRNA to cDNA the Super Script III reverse transcriptase kit (Invitrogen) was applied and qRT-PCR analysis was performed on the CFX96 Touch Real-Time PCR Detection System (BioRad) using SsoFast EvaGreen Supermix (BioRad). Expression levels were normalized to the housekeeping gene glyceraldehyde 3-phosphate dehydrogenase (*GAPDH*) using BioRad software.

qRT-PCR primer sequences:

PARK2 forward primer: TTTCCCAGTGGAGGTCGATT
PARK2 reverse primer: CAAGAACGGCCGCCAAG
ACTB forward primer: CTGGACTTCGAGCAAGAGATG
ACTB reverse primer: GGAAATGAGGGCAGGACTTAG
GAPDH forward primer: GAAATCCCATCACCATCTTCCAGG
GAPDH reverse primer: GAGCCCCAGCCTTCTCCATG
OCT4 forward primer: AGTGAGAGGCAACCTGGAGA
OCT4 reverse primer: GTGAAGTGAGGGCTCCCATA
EN1 forward primer: GTGTCTGCCCACCTCTTCTC

EN1 reverse primer: GCAGTCTGTGGGGTCGTATT
LMX1A forward primer: AGAGCTCGCCTACCAGGTC
LMX1A reverse primer: GAAGGAGGCCGAGGTGTC
GIRK2 forward primer: CCAGTCGGCTGGAATTGAGT
GIRK2 reverse primer: ATGAGAGTGGAGATGCCTGC

Desalting of mass spectrometry samples
A small plug of C_{18} material from a 3M Empore^{TM} disk (Sigma) was inserted in the constricted end of a P200 pipette tip and 1.5 cm of the tip was packed with reversed-phase resin material consisting of a 1:1 mix of Poros 50 R2 (Applied Biosystems) and Oligo R3 (Applied Biosystems) dissolved in 100% acetonitrile (ACN, Sigma) by applying air pressure with a 1 ml syringe. The acidified samples were loaded onto the first micro-columns and washed with 0.1% TFA. The peptides were eluted using 60% ACN/0.1% TFA and the samples were dried by speed vacuum centrifugation.

Enrichment of phosphorylated peptides
Peptides were dissolved in 80% ACN/5%TFA with 1 M glycolic acid (Sigma) and incubated with 0.6 mg titanium dioxide (TiO_2) beads (Titansphere 10 μm, GL Sciences) per 100 μg peptide for 30 min at RT with vigorous shaking. The beads were centrifuged briefly and the supernatant transferred to a new tube with 0.3 mg TiO_2 beads per 100 μg peptide. After 15 min incubation at RT with vigorous shaking and a brief centrifugation the supernatant was collected. The beads were subsequently washed with 80% ACN/1% TFA and 10% ACN/0.1% TFA. The supernatant with the unbound TiO_2 fraction and the washing fractions, both containing the non-phosphorylated peptides, were combined. The phosphorylated peptides were eluted from the beads by incubation with 1.5% ammonium hydroxide solution (Sigma), pH 11.3, for 15 min at RT with vigorous shaking. The beads were spun down and the supernatant passed through C_8 material from a 3M Empore^{TM} disk (Sigma) before the samples were dried.
All the eluates were dried and desalted on homemade columns as described previously, using only R3 material for the phosphorylated peptides and R2/R3 material for non-modified peptides.

Hydrophobic interaction liquid chromatography (HILIC) and high pH fractionation
The non-modified sample was first diluted in 0.1% TFA and approximately 50 μg peptide was fractioned. All phosphorylated peptides were fractionated. The samples were dissolved in 90% ACN, 0.1% TFA (solvent B) and loaded onto an in-house packed TSKgel Amide-80 (Tosoh Bioscience) micro-capillary column (450 μm OD x 320 μm ID x 17 cm) using an Agilent 1200 Series HPLC (Agilent). Peptides were separated using a gradient from 100–60% solvent B (A = 0.1% TFA) running for 30 min at a flow-rate of 6 μl/min. Fractions were collected every 1 min and combined into 12-15 final fractions based on the UV chromatogram and subsequently dried by vacuum centrifugation.

Immunofluorescence staining
Cells cultured in 24-well plates (Corning) containing coverslips were fixed for 15 min at room RT in 4% (w/v) paraformaldehyde (PFA, Sigma) in 0.15 M phosphate buffer (potassium dihydrogen phosphate (Merck) and disodium phosphate (Merck)), pH 7.4, and rinsed with 0.05 M Tris-Buffered Saline (TBS, Sigma), pH 7.4, with 0.1% Triton-X-100 (Sigma). Cells were permeabilised and unspecific binding blocked with TBS containing 10% goat- (Millipore), donkey- (Millipore) or sheep serum (Sigma) according to the host of the secondary antibodies, and incubated over night (ON) at 4 °C with primary antibodies diluted

in TBS/10% donkey or sheep serum. Cultures were rinsed in TBS/0.1% Triton-X-100 and incubated with Alexa Fluor 555 goat anti-rabbit IgG (Abcam #150078) or Alexa Fluor 488 goat anti-rabbit IgG (Invitrogen # A11008) 1:500 in TBS/10% goat serum for 2 h at RT. Cell nuclei were counterstained with 10 μM 4",6-diamidino-2-phenylindole dihydrochloride (DAPI, Sigma) in TBS. Cultures were mounted onto glass slides with ProLong Diamond mounting medium (Molecular Probes).

Western blotting procedure

Cell pellets were lysed in RIPA buffer (50 mM Tris-hydrochloride (Sigma), 150 mM sodium chloride (Sigma), 1% Tergitol-type NP-40 (Sigma), 0.5% sodium cholate hydrate (Sigma), and 0.1% SDS (Sigma), pH 8) containing phosphatase (PhosSTOP tablets, Roche) and protease inhibitor (Complete Tablets, Roche) and sonicated for 10 secs at 10 amplitude microns on ice. Protein concentrations were determined with bicinchoninic acid (BCA) protein assay (ThermoFisher) and equal amounts of protein from each sample were denatured for 5 min at 95 °C in 6X Laemmli buffer. Proteins were separated on a 4-15% precast gel (Biorad) at 200 V with running buffer (35 mM SDS, 250 mM Trizma base (Sigma), and 1,92 M glycine (Sigma), pH 8.3) and transferred to a polyvinylidene difluoride (PVDF) membrane (Biorad) at 25 V, 2.5 A for 11 min using the Trans-Blot Turbo Transfer System (Bio-Rad). MagicMark XP Western Protein Standard (ThermoFisher) and BLUeye Prestained Protein Ladder (Geneflow) were used to estimate the molecular weight of the proteins. Membranes were blocked for 2 hrs at RT in 5% (w/v) skim milk (Sigma) diluted in Tris-buffered saline (20 mM Tris-hydrochloride and 150 mM sodium chloride) with 0.1%Tween 20 (TBST, Sigma) with gentle shaking. Primary antibodies were diluted in 5% (w/v) skim milk in TBST and membranes incubated ON at 4 °C with gentle shaking.

Blots were washed four times with TBST and incubated with horseradish peroxidase (HRP)-conjugated goat anti-mouse or -rabbit IgG (Bio-Rad), diluted 1:5000 in 5% (w/v) skim milk in TBST, for one hr at RT with gentle shaking. After being washed, the membranes were imaged using ECL (Millipore) on a ChemiDoc Touch Imaging system (Bio-Rad), and the optical density of each band was quantified using Image Lab software (Bio-Rad). The protein expression level for each lane was normalized to the intensity of the corresponding β-actin band. Subsequently, the protein expression levels of *PARK2* KO cells were normalised to levels of the control in the same differentiation to obtain relative expression levels for each differentiation. For validation of proteomic results, relative protein expression levels were determined from at least 2-3 subsequent differentiations, independent from the ones used for the proteomic analysis.
